# Unusual spontaneous intraperitoneal hemorrhage: three case reports

**DOI:** 10.1093/jscr/rjae475

**Published:** 2024-08-03

**Authors:** Yurii Lovitskyi, Yaryna Romanenko, Maksym Shcherbyna, Kristina Zadorozhna, Roman Kalyna, Evhen Herasymenko, Kostiantyn Kopchak

**Affiliations:** Department of Surgery, Medical Network “Dobrobut”, Idzikovskih, 3, Kyiv 2100, Ukraine; Department of Surgery, Medical Network “Dobrobut”, Idzikovskih, 3, Kyiv 2100, Ukraine; Department of Surgery, Medical Network “Dobrobut”, Idzikovskih, 3, Kyiv 2100, Ukraine; Department of Surgery, Medical Network “Dobrobut”, Idzikovskih, 3, Kyiv 2100, Ukraine; Department of Surgery, Medical Network “Dobrobut”, Idzikovskih, 3, Kyiv 2100, Ukraine; Department of Surgery, Medical Network “Dobrobut”, Idzikovskih, 3, Kyiv 2100, Ukraine; Department of Surgery, Medical Network “Dobrobut”, Idzikovskih, 3, Kyiv 2100, Ukraine

**Keywords:** spontaneous hemoperitoneum, greater omentum arterio-venous malformation, pathological splenic rupture, idiopathic omental bleeding, gastric intramural hematoma, atraumatic splenic rupture

## Abstract

Introduction and importance: Spontaneous hemoperitoneum (SH) is a rare, life-threatening condition characterized by nontraumatic and non-iatrogenic intraperitoneal bleeding. This article explores three unique cases of SH, shedding light on unusual causes and emphasizing the critical role of diagnostic imaging and exploratory laparotomy in management. Methods: The study was a retrospective single-center non-consecutive case series. Results: We report three distinct cases of SH, each originating from uncommon sources: rupture of greater omentum arterio-venous malformation, a branch of the left gastric artery, and pathological splenic rupture. Clinical evaluation, diagnostic imaging, and surgical interventions are detailed for each case. Conclusion: These rare cases underscore the diverse etiologies of SH, including idiopathic omental bleeding, gastric intramural hematoma, and atraumatic splenic rupture. Enhanced CT imaging plays a crucial role in diagnosis, enabling the characterization of underlying pathologies. Exploratory laparotomy proves to be an essential treatment option for unstable patients with suspected or confirmed diagnoses of SH.

## Introduction and importance

Spontaneous hemoperitoneum (SH) is a rare life-threatening condition. It is defined as nontraumatic and non-iatrogenic etiology of blood within the peritoneal cavity. Common sources of spontaneous intraperitoneal bleeding are visceral, gynecologic, coagulopathy-related, and vascular. It commonly presents with signs of acute intraperitoneal bleeding, abdominal compartment syndrome and hemorrhagic shock in severe cases [1].

When gynecologic causes are not considered, the most common cause of SH is liver pathology, such as large hemangiomas, adenomas, focal nodular hyperplasi,a and malignant hepatic lesions. The second most common solid organ to lead to SH is spleen, usually by pathological splenic rupture or vascular causes, includes aneurysms, pseudo-aneurysms, or arterial dissection. Other possible rare causes of SH include hemorrhage from a highly vascular neoplasm, bleeding from a vascular lesion, anticoagulation therapy, blood dyscrasias [2].

It is critical to identify the clinical symptoms of hemorrhage and treat any active bleeding promptly. Initial therapy in the hemodynamically stable patients is endoscopic technique like angiography and embolization. But, in the cases of unresponsive hemorrhagic shock or failure of interventional techniques, surgery should be considered [3].

We present here three rare cases of SH due to rupture of greater omentum arterio-venous malformation, branch of left gastric artery, and pathological splenic rupture.

## Methods

The study was a retrospective single-center non-consecutive case series, which was carried out at the Medical Center “Dobrobut”, Kyiv, Ukraine.

We report three distinct cases of SH, each originating from uncommon sources: rupture of greater omentum arterio-venous malformation, a branch of the left gastric artery, and pathological splenic rupture. Clinical evaluation, diagnostic imaging, and surgical interventions are detailed for each case.

The work has been reported in line with the PROCESS criteria. This work has been reported in line with the SCARE criteria.

## Results

### Case presentation 1

A 54-year-old male patient was referred to the emergency department with severe general weakness and persistent left upper abdominal pain, with 6-hour evolution. On examination, the patient was hemodynamically unstable. The abdomen was moderately distended with signs of peritoneal irritation. The patient had no prior medical history, with hemoglobin of 10.9 g/dl and arterial blood pressure of 90/60 mm Hg.

**Figure 1:**
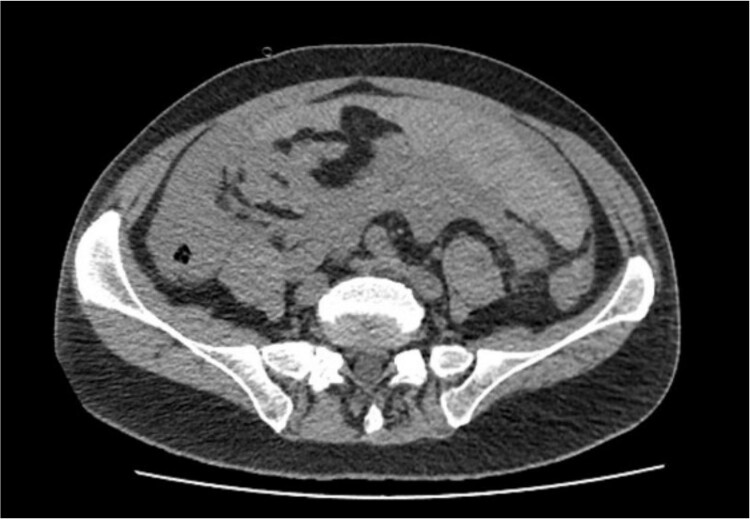
Computed tomography image on admission: moderate ascites with organized clot.

Non-contrast abdominal computed tomography (CT) revealed hemoperitoneum: moderate ascites with organized clot on the surface of greater omentum (Fig. 1)

After clinical case evaluation and management options discussion with the patient, informed consent was obtained for an exploratory laparotomy. On laparotomy, around 1.5 l of haemorrhagic fluid and a 500-ml clot was removed from the peritoneal cavity. A hemoperitoneum originated in a 7 × 5 cm^2^ tumor lesion of the greater omentum. Part of greater omentum with tumor was resected. Histopathology report confirmed the mass to be an arteriovenous malformation. In the post-operative period, the patient needed a blood transfusion, although there was no evidence of blood loss. The patient was discharged on the seventh post-operative day without complications.

### Case presentation 2

The second case is a 51-year-old male patient who was admitted to the emergency department with abdominal pain, dizziness, nausea, and recurrent vomiting with a 12-h evolution. The patient had a medical history of chronic fibrinous-degenerative pancreatitis, pseudocysts of the pancreas. Two months ago, he was submitted to laparoscopic drainage of walled-off pancreatic fluid collection.

**Figure 2:**
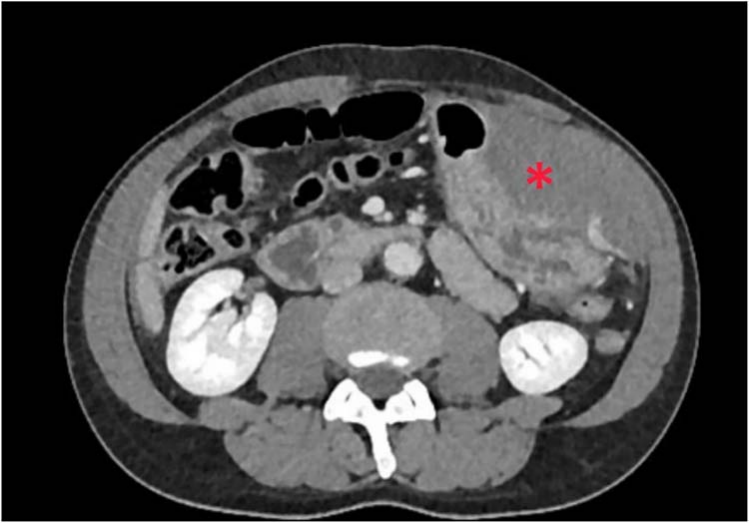
Intramural hematoma in anterior stomach wall.

**Figure 3:**
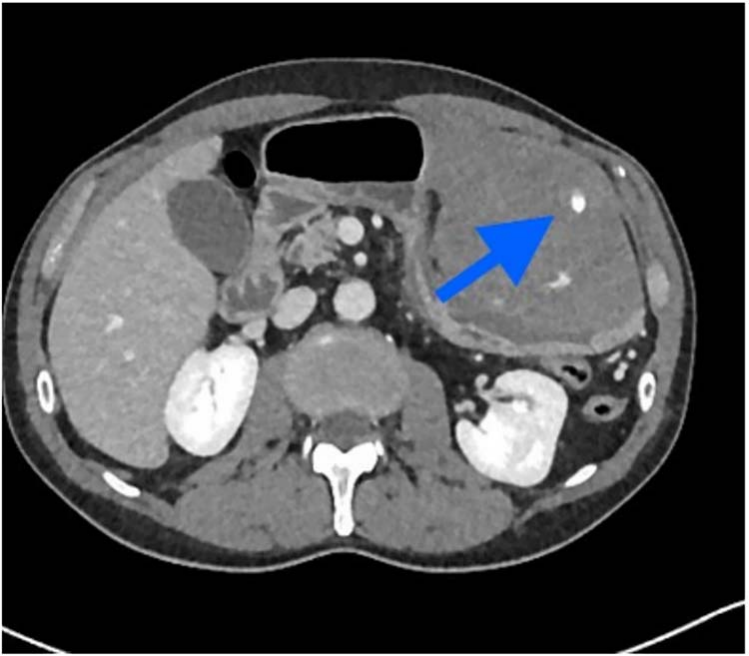
Contrast extravasation in hematoma.

The patient was pale, hemodynamically stable, and with signs of peritoneal irritation. His hemoglobin was 15.1 g/dL. Contrast enhanced CT demonstrated 11.6 × 8.8 × 16.5 cm^3^ intramural hematoma in anterior stomach wall with active bleeding and hemoperitoneum (Figs 2 and 3). Exploratory laparotomy was done after optimizing the patient.

**Figure 4:**
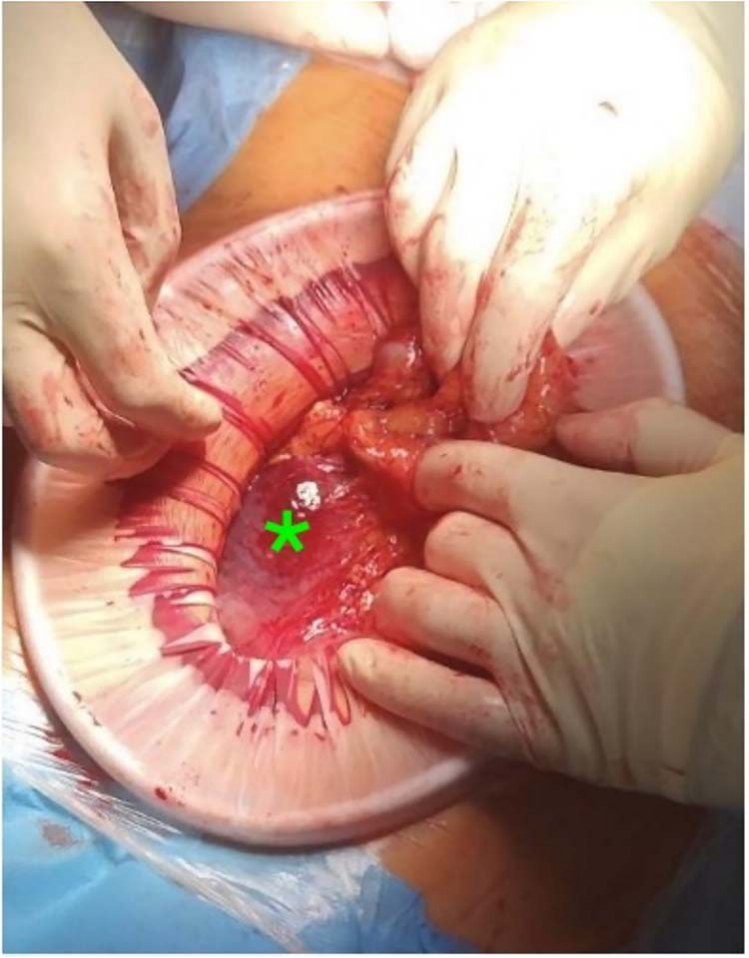
Intramural hematoma in anterior stomach wall.

**Figure 5:**
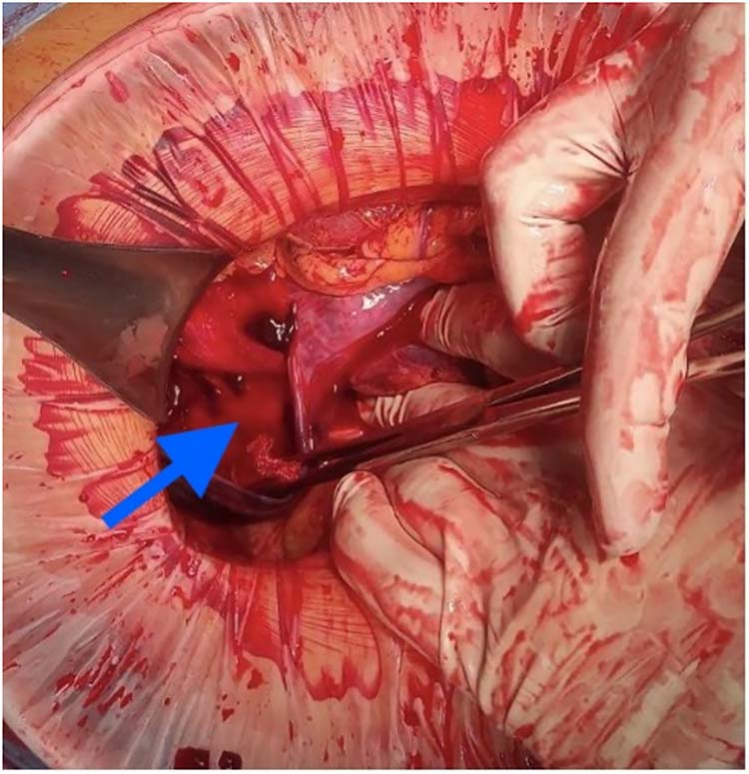
Active bleeding in the hematoma cavity.

At laparotomy, it was apparent that a large hematoma had occupied the anterior aspect of the stomach causing extensive serosal stretch from fundus to pylorus (Fig. 4). Serous membrane under hematoma was ruptured and about 500 ml of hemorrhagic liquid with clots was detected in the peritoneal cavity. Serous membrane rupture was widened, evacuated blood clots and revealed small branch of left gastric artery as origin of bleeding (Fig. 5). Suture ligation of artery was performed. Surgery finished by drainage of abdominal cavity, cavity of hematoma with serous membrane suturing, and microjejunostomy for post-operative nutritional support.

The etiology of the hematoma was not proven by pathological findings. He was admitted to the general surgical ward after surgery and discharged on the eighth post-operative day. Microjejunostomy tube was removed on the 30th post-operative day without complications.

### Case presentation 3

**Figure 6:**
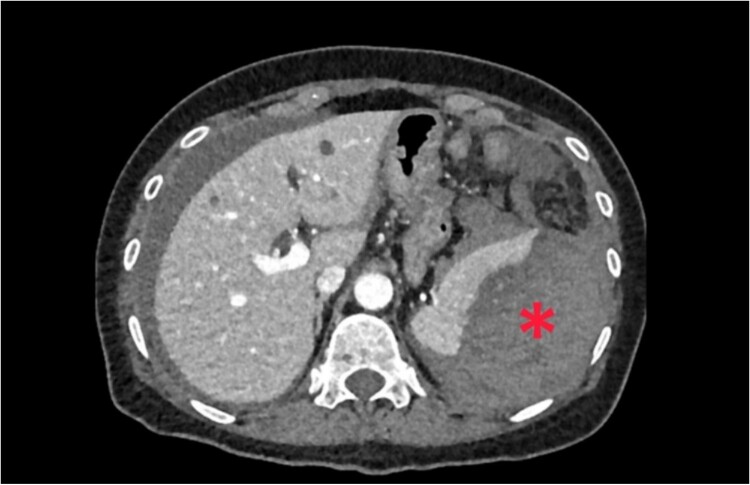
Large perisplenic hematoma.

A 62-year-old woman admitted at the emergency department complaining of abdominal pain with sudden-onset 6 h ago. On arrival, there was a collapse, with a blood pressure of 80/60 mmHg and a pulse of 120/min. She was pale, apyretic, and had with signs of peritoneal irritation. The hemoglobin was at 7.3 g/dl. After resuscitation measures and transfusion of 2 units of red blood cells transfusions, contrast enhanced CT was performed. CT showed free fluid in the abdomen and a large perisplenic hematoma (Fig. 6). As the patient remained haemodynamically unstable, she underwent an exploratory laparotomy. During laparotomy, there was a hemoperitoneum related to complete decapsulation of spleen. Splenectomy was performed. Histological examination confirmed the non-pathological aspect of a decapsulated spleen. The patient’s hospital course was uncomplicated. She was discharged on the seventh post-operative day.

## Clinical discussions

Idiopathic omental bleeding is considered one of the causes of SH. Spontaneous omental bleeding is a life-threating condition, with a mortality rate exceeding 30% [4]. Several causes of spontaneous omental bleeding (neoplasia, arterial aneurysm, vasculitis, and anticoagulant therapy, administered sildenafil citrate) have been reported [5]. Common signs of idiopathic omental bleeding are abdominal pain and distension, tachycardia, hypotension, abdominal compartment syndrome in severe cases [2]. Some cases of omental bleeding can mimick appendicitis or peritonitis before surgery [6, 7].

Contrast enhanced CT is the most effective imaging tool since signals correspond to intraperitoneal bleeding [3]. The surgical option is suitable in patients with persistent hypotension or unconfirmed preoperative diagnosis. The reason why surgery is often needed is that few cases are diagnosed correctly before treatment [8].

Gastric intramural hematoma is a rare condition. A review of the literature suggests that the most common aetiology is coagulopathy, related to the use of anticoagulation, haemophilia, or myelofibrosis [9]. Other causes include peptic, vascular aneurysms, spontaneous, and idiopathic cases [10]. The most specific and sensitive in diagnosing intramural hematomas is contrast-enhanced CT [11]. Commonly, in patients with coagulopathies, conservative treatment is used, with administration of clotting factors and blood transfusions [12]. If conservative treatment fails, hemostasis includes arterial embolization, endoscopic or percutaneous drainage, and surgery [13]. Surgical management of gastric intramural hematoma is recommended where there is unclear diagnosis, suspected complications, involving a large part of the gastric wall, with ongoing bleeding [14].

Atraumatic splenic rupture is rare condition, associated with several underlying infectious, gastrointestinal, haematological, and systemic pathologies [15]. In the absence of trauma, imagine tools are necessary to confirm diagnosis. CT scan is most specific and sensitive for diagnosing splenic rupture. The most common finding on CT is splenomegaly with splenic lacerations and intraperitoneal or subcapsular bleeding [16]. Low-grade injuries (I–II) may be managed conservatively, whereas higher-grade (IV–V) injuries generally require surgery. In the cases, when the patient is haemodynamically unstable, surgery is necessary [17].

## Conclusions

SH is a rare condition. We report three extremely rare cases of patients with an SH, caused by rupture of greater omentum arterio-venous malformation, branch of left gastric artery, and pathological splenic rupture.

Imaging, especially enhanced CT, plays a critical role in the diagnosis of hemoperitoneum and can detect and characterize the underlying pathology.

Explorative laparotomy is necessary treatment option for unstable patients with suspected of confirmed diagnosis of SH.

## Patient consents

Consent to publish the case report was not obtained from patient. This report does not contain any personal information that could lead to the identification of the patient. Publication has been authorized by the deanery of the faculty of medicine and the administration of our hospital.
